# Guidelines on acute gastroenteritis in children: a critical appraisal of their quality and *applicability *in primary care

**DOI:** 10.1186/1471-2296-12-134

**Published:** 2011-12-02

**Authors:** José van den Berg, Marjolein Y Berger

**Affiliations:** 1 Department of General Practice, Erasmus Medical Center, Erasmus University Rotterdam, the Netherlands; 2 Department of General Practice, University Medical Center Groningen, University of Groningen, the Netherlands

## Abstract

**Background:**

Reasons for poor guideline adherence in acute gastroenteritis (AGE) in children in high-income countries are unclear, but may be due to inconsistency between guideline recommendations, lack of evidence, and lack of generalizability of the recommendations to general practice. The aim of this study was to assess the quality of international guidelines on AGE in children and investigate the generalizability of the recommendations to general practice.

**Methods:**

Guidelines were retrieved from websites of professional medical organisations and websites of institutes involved in guideline development. In addition, a systematic search of the literature was performed. Articles were selected if they were a guideline, consensus statement or care protocol.

**Results:**

Eight guidelines met the inclusion criteria, the quality of the guidelines varied. 242 recommendations on diagnosis and management were found, of which 138 (57%) were based on evidence.

There is a large variety in the classification of symptoms to different categories of dehydration. No signs are generalizable to general practice.

It is consistently recommended to use hypo-osmolar ORS, however, the recommendations on ORS-dosage are not evidence based and are inconsistent. One of 14 evidence based recommendations on therapy of AGE is based on outpatient research and is therefore generalizable to general practice.

**Conclusions:**

The present study shows considerable variation in the quality of guidelines on AGE in children, as well as inconsistencies between the recommendations. It remains unclear how to asses the extent of dehydration and determine the preferred treatment or referral of a young child with AGE presenting in general practice.

## Background

In acute gastroenteritis (AGE), a common childhood illness occurring worldwide, the most threatening complication is dehydration. In high-income countries, implementation of oral rehydration therapy (ORT) has not been as successful as in low-income countries[1,2]. In Dutch general practice, ORT is prescribed in only 4% of children presenting with acute diarrhea [3]. It is thought that too many children are referred to hospitals and that a significant number of the hospital admissions can be avoided [4,5]. There is a lack of evidence for the use of antibiotics, antiemetics and antidiarrheals in AGE, and serious side-effects of loperamide in young children have been reported [6,7]. Nevertheless, of children presenting with acute diarrhea in Dutch general practice, 16% receive a prescription for antiemetics, antibiotics or antidiarrheals[3].

Implementation of a guideline on AGE management in children can lead to a reduction in invasive procedures and hospital admissions, and shorter hospital stays [4,8,9]. Because guideline quality is important, reports are available to assist in developing, appraising [10] and using [11,12] a guideline and its recommendations. An up-to-date high-quality guideline generally means that potential biases of guideline development have been addressed, and that the recommendations are practicable, reflect the balance between desirable and undesirable effects, incorporate quality of evidence, consider variability in values of benefits, risks, inconveniences, and costs [10-12].

However, although physicians consider guidelines to be helpful, negative attitudes about the goal of the guideline, the producers of the guideline, the practicality of the guideline, as well as controversies and/or inconsistencies between guidelines regarding recommendations, can reduce adherence [13-16]. The reasons for poor guideline adherence in AGE in children in high-income countries are unclear, but may be due to inconsistency between guideline recommendations, lack of evidence, and a lack of generalizability of the recommendations to general practice [13].

The aim of the present study is to assess the quality of international practice guidelines for the management of acute diarrhea in children in high income countries with the Appraisal of Guidelines, Research and Evaluation (AGREE) instrument. We aim to investigate the consistency of the recommendations, evaluate the quality of evidence and the practicability of these recommendations to general practice, where most children with AGE initially present.

## Methods

### Identification of guidelines

To identify guidelines on acute diarrhea in children, searches were made in websites of professional societies in the clinical fields of paediatrics, gastroenterology and general practice, and websites of institutes involved in guideline development. On each website we used the available search technique, searching for diarrhea and/or gastroenteritis. In addition, a systematic search of the literature was performed using Medline, Embase, Cinahl and the Cochrane Library database. The search term combination captured the concept diarrhea, practice guidelines and children, using a variety of index terms, text words and word variants. No limitations were used (see Appendix 1).

### Selection of guidelines

Clinical practice guidelines were defined to be "systematically developed statements to assist practitioner and patient decisions about appropriate healthcare for specific clinical circumstances" [17,18]. Selection of articles was performed using the following pre-defined selection criteria: i.e. articles were selected if they were: a guideline, consensus statement or care protocol on the management of acute diarrhea in children, and were produced by a professional organisation (i.e. an organisation that is the official representative of an important usergroup (paediatricians, gastro-enterologists or general practitioners). Guidelines were excluded if they were: opinion based, not designed for children, designed for low-income countries or exclusively designed for children with severe co-morbidities.

Selection of articles was performed by two independent researchers. Disagreements about inclusion or exclusion were resolved by consensus.

### Appraisal of guideline quality

The quality of a guideline was assessed using the Appraisal of Guidelines for Research and Evaluation (AGREE) instrument [10]. The AGREE instrument contains 23 items grouped in six domains - 1/scope and purpose, 2/stakeholder involvement 3/rigour of development; 4/clarity and presentation; 5/applicability 6/editorial independence - and one overall assessment item, judging whether the guideline ought to be recommended for its use in clinical practice. To evaluate each item within the domains a four-point Likert scale is used, ranging from strongly disagree to strongly agree (1 to 4). For the overall judgment a three-point scale is used ranging from not recommended to strongly recommended. (See Appendix 2). Two reviewers independently assessed the quality of each guideline. MB is a general practitioner (GP) and professor of General Practice at the university hospital Groningen, JB is a GP and researcher at the Erasmus MC. After the initial assessment, items with a difference in score of more than two points were discussed in a consensus meeting and the scores were adjusted if consensus was reached. Items with a difference in score of one point were considered to be comparable.

### Extraction of recommendations

Data were extracted using a predefined form, covering the following items: 1) general information: guideline developers; GP in the development team (yes/no); method used for assessing the level of evidence, i.e. the ranking system used to stratify evidence by quality, for which multiple systems have been developed [19-24], 2) recommendations: all recommendations for diagnosis and management with their accompanying level of evidence (LofE), and 3) in order to estimate applicability for primary care for all evidence used to formulate a recommendation, study type, setting and population of each reference were investigated by appraising abstracts or full-text articles. One researcher (JB) collected the data and another (MB) verified the data.

### Consistency of recommendations

All recommendations were grouped by subject and the number of guidelines making a recommendation on a subject was summed. If more than 50% of these guidelines made an identical recommendation, the recommendation was considered consistent.

### Generalizability to general practice

For recommendations based on published evidence, an appraisal about the generalizability to general practice was made. Recommendations were defined as applicable to general practice if the evidence came from studies in general practice or an outpatient non-referred population. A recommendation was not applicable to general practice if it was based on evidence emerging from studies in a hospital setting or a referred population. If evidence came from a mixed population (outpatient as well as inpatient), it was assessed if a separate analysis was performed to evaluate differences in outcome between in- and outpatient populations, if this was not the case, generalizability was considered inconclusive.

For recommendations based on consensus, recommendations were said to be applicable to general practice if a GP was involved in guideline development. The assumption was that the GP would have controlled for feasibility and applicability of the recommendation in general practice.

### Diagnosis of dehydration

Without predefined definition most guidelines classified the extent of dehydration differently. Therefore, we defined three subgroups based on the WHO definition [25]: no (<3% dehydration), mild-moderate (3-9% dehydration) and severe (>9% dehydration). In accordance with the classification used in the guideline, the reviewers assigned signs and symptoms to one of these categories.

## Results

### Identification and selection of guidelines

**Figure 1:**
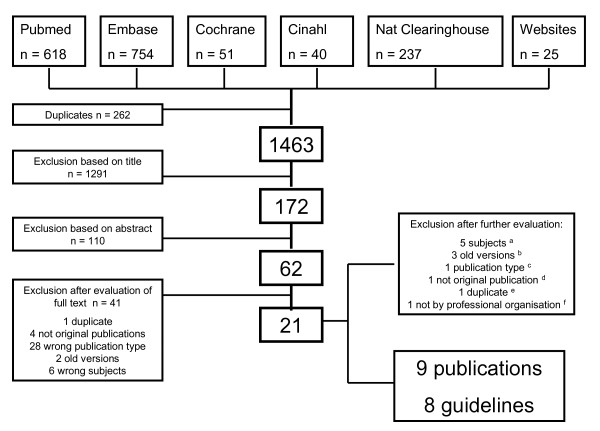
**Flowchart of the inclusion/exclusion process**. A ^24 26-27 33 41 ^B ^25 36 38 ^C ^40 ^D ^39 ^E ^23 ^F ^37^

A total of 1,725 titles were found in the search; after exclusion of duplicates (n = 262) and screening of titles and abstracts, 62 citations were suitable for full-text evaluation. After the initial appraisal, 21 citations were evaluated in detail; one additional duplicate was found [21,25-43]. Nine documents [21,25,30-34,36,37], forming 8 guidelines, met our selection criteria (Figure 1, Table 1).

**Table 1:** Characteristics of the eight guidelines included in the present study.

Author/Organisation	Country of development	Year of publication	Intended users	Level of evidence stated?	GP in development team?	No. of key recommendations	No. of recommendations on diagnosis and management	No. of recommendations (partly) based on evidence
Armon, Stephenson, MacFaul 2001 (ARM) [34]	UK	2001	Hospital care	Yes*	No	9	13	8
Centers for Disease Control and Prevention (CDC) [25]	USA	2003	Not stated	Not stated	No	Not stated	35	14
European Society for Paediatric Gastroenterology, Hepatology and Nutrition European Society for Paediatric Infectious Diseases (ESP) [37]	Europe	2008	All levels of care	Yes*	No	10	74	47
Canadian Paediatric Society (CPS) [30]	Canada	2006	Not stated	Yes^¥^	No	9	17	11
World Gastroenterology Organisation (WGO) [32]	Worldwide	2008	All levels of care	Not stated	No	Not stated	21	Not stated
Nederlands huisartsengenootschap (NHG) [31]	The Netherlands	2007	General practitioners	Not stated	Yes	Not stated	21	15
Cincinnati Children's Hospital Medical Center (CCH) [21]	USA	2006	Hospital care	Yes^ ± ^	No	17	26	22
National Institute for Health and Clinical Excellence (NICE) [33]	United Kingdom	2009	All levels of care	Yes^§^	Yes	8	35	21

* Muir-Gray system[19]

**^¥ ^**System created by the Canadian Taskforce on preventive health [20]

^ ± ^CCHMC evidence grading scale [21]

**^§ ^**Scottish guideline development group system [22].

### Guideline characteristics

Table 1 lists the characteristics of the included guidelines. A GP was part of the guideline development team in two guidelines: NICE and NHG.

### Guideline quality

The quality of the guidelines was heterogeneous, ranging from poor to excellent quality (Table 2). The NICE guideline scored > 50% on all items [33], and the CDC scored below 40% on all items [30]. All but one guideline (CDC) scored high (> = 50%) on clarity and presentation. Lowest scores were given for 'applicability' (23.6%). Only the NICE guideline scored high on this latter item (88.9%). The three guidelines that would not be recommended by the reviewers scored below 40% on all items, except clarity and presentation.

**Table 2:** Domain scores (in %) after AGREE assessment of the eight guidelines^ ± ^

	Scope and purpose	Stakeholder involvement	Rigour of development	Clarity and presentation	Applicability	Editorial independence	Recommend Guideline*
ARM	100	54.2	88.1	66.7	38.9	50.0	U
CDC	27.7	12.5	35.7	33.3	16.7	8.3	WNR/U
ESP	94.4	37.5	90.5	75.0	11.1	100	SR/R
CPS	16.7	20.8	38.1	70.8	5.6	16.7	WNR
WGO	33.3	33.3	28.6	66.7	16.7	0	WNR
NHG	33.3	25.0	66.7	91.7	11.1	58.3	R
CCH	83.3	57.7	85.7	83.3	0	100	R
NICE	88.9	75.0	88.1	95.8	88.9	100	SR

^ ± ^Maximum possible score = 100%

* Researchers recommendation for guideline use:

SR = strongly recommend

R = recommend

WNR = would not recommend

U = unsure

### Number of recommendations

In total, 242 recommendations on diagnosis and management were found, of which 138 (57%) were based on evidence (Table 1). Information upon the number and the percentage of studies that corroborate for each evidence based recommendation is given in table 3. Five guidelines used key recommendations [21,30,33,34,37]. The ESPGHAN guideline made the most recommendations (n = 74), and had the most recommendations that were based on evidence (n = 47). The CPS had lowest number of recommendations (n = 17). The WGO guideline did not state the source of evidence used to make a recommendation.

**Table 3:** Assessment of dehydration status and recommendations on therapy.

	No dehydration	Mild to moderate dehydration	Severe dehydration	Consistent?Yes/No (№/Total)	Setting
	Normal mental status*^#∞§ ^	Normal to altered mental status*^#∞§ ± ^	Normal, altered to comatose mental status *^# $ ∞ ± Φ^	Y (7/8)	Hospital^ ± ∞^
	Normal*^# ^to slight increase in thirst^#^*^∞$^	Thirsty ^#∞*$^	Greatly increased thirst or drinking poorly or not at all^$^*^#∞^	N (4/8)	Hospital^∞^
	Normal to slightly elevated heart rate^#∞$§ ^	Heart rate normal to increased ^#∞$§Φ^	Tachycardia, (with bradycardia in most severe cases)^#$ ∞§^	Y (5/8)	Hospital ^∞Φ^
	Normal pulse quality^#∞§^	Quality of pulses normal^§ ^to decreased ^#∞ΦΨ^	Weak, thready or impalpable pulses^#∞§^	Y (7/8)	Hospital ^∞ΦΨ^
	Normal breathing^∞§^	Normal, fast or deep (acidotic^ ± ^) breathing ^#§Φ Ψ^	Deep (acidotic^ ± ^) breathing ^#Φ§ ± ^	Y (5/8)	Hospital ^ΦΨ ± §^
	No sunken eyes*^#∞§^	Sunken eyes ^±#∞*$§Φ^	(deeply) sunken eyes*^$ ∞# ± ^	Y (7/8)	Hospital ^ ± Φ∞§^
**Diagnosis**	Normal/present tears^#∞^	Decreased^ ± # ^or absent ^ΦΨ ^tears^∞^	Absent tears^#∞ ± ^	Y (5/8)	Hospital ^ ± Φ∞^
	Moist^#§ ^to slightly dry^∞$ ^mucous membranes	Dry mucous membranes^# ± ∞$§Φ^	Very dry mucous membrane^#$ ∞^	Y (6/8)	Hospital ^ ± Φ∞§^
	Immediate skin pinch*^#§^	Skin pinch in 1- 2 seconds (decreased) ^# ± ^*^∞$§ΦΨ^	Very low skin pinch (>2 seconds)*^∞#$ ± ^	Y (8/8)	Hospital ^Ψ ± Φ§^
	Normal capillary refill^#∞§^	Normal^§ ^to prolonged capillary refill ^#∞ΦΨ^	Prolonged or minimal capillary refill^#∞§^	Y (5/8)	Hospital ^ΨΦ§∞^
	Warm extremities^#§^	Warm^§ ^to cool extremities ^#ΦΨ^	Cold, mottled or cyanotic extremities^# $ ± §^	Y (6/8)	Hospital ^Ψ ± Φ^
	Urine output normal^#§ ^to slightly decreased^∞$^	Decreased urine output^#∞$§^	Minimal (to no) urine output^#$∞^	N (4/8)	Hospital ^∞^
	"No signs"^ ± ^	Normal blood pressure^∞§^	Hypotension/circulatory collapse^$ ± ∞§^	N (4/8)	Hospital ^∞^
	Normal anterior fontanel^∞^	Sunken anterior fontanel ^∞$Φ^	Very sunken anterior fontanel^$∞^	N (3/8)	Hospital ^∞Φ^

					

	(Premixed) reduced osmolarity or hypotonic ORS should be used for rehydration*^#$ ± §ΦΨ^			Y (7/8)	In/outpatient ^$± §ΦΨ^
	Continue breastfeeding throughout rehydration ^Φ ± §Ψ$∞ #^*			Y (8/8)	In/outpatient ^Φ ± §Ψ^
	Start age-appropriate diet during^∞ ^or after initial rehydration ^Φ ± §Ψ$#^*			Y (7/8)	In/outpatient ^Φ ± §Ψ$∞#^
	High sugar beverages, canned or carbonated drinks should be avoided ^#ΦΨ$§^*			Y (6/8)	Hospital ^§Ψ^
	Dilution of formula and the use of milk-free formula is unnecessary ^Φ ± §Ψ$#^*			Y (7/8)	Hospital ^§∞Ψ$^
	Antiemetics are discouraged *^#Φ∞§Ψ^			Y (6/8)	In/outpatient ^Ψ§Φ^
	Racedotril is not (yet) recommended ^Φ§#^			N (3/8)	Hospital ^Φ§#^
**Therapy**	Racedotril may be useful^∞Ψ^			N (2/8)	Hospital ^∞Ψ^
	Ondansetron is not (yet) recommended ^Φ§#^			N (3/8)	Hospital ^Φ§^
	Antidiarrheals should not be used/have no benefit*^Φ∞Ψ§ ± ^			Y (6/8)	In/outpatient ^ΦΨ§ ∇^
	Zinc supplementation is not (yet) recommended for developed countries^Ψ§#^			N (3/8)	In/outpatient ^§Ψ^
	Probiotics can be used/considered ^∞Ψ ^/do not have to be discouraged ^Φ^			N (3/8)	In/outpatient ^∞Ψ Φ^
	Probiotics are not recommended ^§^			N (1/8)	In/outpatient ^§^
	Prebiotics are not recommended ^Ψ^			N (1/8)	Outpatient ^Ψ^

** Number of guidelines mentioning this symptom in at least one subgroup

*WGO ^# ^CDC ^$^CPS ^ ± ^ARM

^∞^CCH ^Φ^NHG ^Ψ^ESP ^§ ^NICE

^∇ ^= research performed in developing countries

Five guidelines stated the LofE of a recommendation. Four ranking systems for the classification of the LofE were used. Two ranking systems (CPS and Muir Gray) combined quality of evidence and strength of the recommendation in one score containing a number and a letter. Two ranking systems classified quality of evidence only. Quality of evidence could be classified on a range of 5 to 12 levels. The best score for quality of evidence was appointed to a systematic review of multiple well designed RCTs (Scottish guideline, Muir Gray); a systematic review (CCHMC) or at least one properly randomized controlled trial (CPS). Because of the heterogeneity of the definition of the classes, we refrained from comparing the outcome of the ranking systems.

### Categories of dehydration

Categories of dehydration were defined differently. The NHG guideline only described the categories 'no' and 'severe' dehydration. The ESPGHAN guideline encouraged the use of categories, but did not define how to categorize dehydration. It did mention, however, the signs for 5% dehydration, and these signs were placed in our mild-moderate group. The remaining guidelines used categories, with (WHO, ARM, CCH) or without (WGO and NICE) percentages, for the extent of dehydration; the signs and symptoms were placed in our categories accordingly.

Table 3 presents recommendations on diagnosis (signs and symptoms) and therapy for dehydration. The consistency of each recommendation is stated, as well as the setting of the research that was used to form the recommendation.

### Recommendations on the diagnosis of dehydration

There were no recommendations for the diagnosis of dehydration based on consensus only. All guidelines provide diagnostic signs/symptoms and relate them to different categories of dehydration. However, there is a large variety in the classification of symptoms to the different categories. ''Altered mental status'; 'normal to increased heart rate'; 'normal to decreased pulses'; 'normal to fast or deep (acidotic) breathing'; 'sunken eyes'; 'decreased or absent tears'; 'dry mucous membranes'; 'decreased skin pinch'; 'normal to prolonged capillary refill' and 'warm to cool extremities' were consistently related to one of the categories of dehydration. But only 'decreased skin pinch' was consistently related to mild-moderate dehydration in all guidelines. Inconsistent recommendations were 'thirst'; 'decreased urine output'; 'blood pressure' and 'sunken fontanel'. The sign 'normal to slight increased thirst' was the only sign consistently mentioned (in > 50% of guidelines) for the subgroup 'no dehydration'. Nine signs were consistently mentioned for the subgroup 'mild to moderate dehydration' and four signs were consistently mentioned for the subgroup 'severe dehydration' (Table 3).

In two guidelines (WGO, CPS), the evidence for the assigned symptoms for assessing dehydration status is not stated. Four guidelines (NHG, ESP, NICE, CCH) use the study of Steiner et al. [44], a meta-analysis on diagnostic signs for dehydration. These guidelines complemented their recommendations by using different additional studies [45-49] (five of which were also included in the above mentioned meta-analysis) and existing guidelines [21,25,34,37]. CDC and ARM used hospital-based cohort studies (both included in the meta-analysis of Steiner et al.) [45,50] or existing guidelines [25,51,52]. Only one complementary study, a cohort study by MacKenzie et al. [45] included in the meta-analysis of Steiner et al. was used in more than one guideline (NHG and ARM). All recommendations were based on research in a hospital setting in referred children, and therefore not applicable to general practice.

### Recommendations on management

In total, 22 recommendations on the management of AGE were made: 14 recommendations were based on evidence and 8 on consensus. Seven recommendations based on evidence and 5 based on consensus are consistent. All guidelines, except one (CCH) recommend that hypo-osmolar rehydration solution should be used to prevent or treat dehydration (evidence based). Table 4 presents recommendations on ORS dosage. Two guidelines, ESP and CCH, did not provide a recommendation for ORS dosage. The remaining 6 guidelines provide three consistent recommendations: children with mild-moderate dehydration should be rehydrated; their ongoing losses should be replaced; and children with severe dehydration should be referred to hospital. There are no consistent recommendations on ORS dosage. Two recommendations are generalizable to general practice (NICE and NHG). None of the recommendations presented in Table 4 are evidence based.

**Table 4:** Oral rehydration solution (ORS) dosages recommended for each dehydration subgroup.

No dehydration	Mild to moderate dehydration	Severe dehydration	Evidence used
			
Prevention of dehydration: ^ ± § Φ^	Rehydration: $*^ ± § Φ^		
• 100 ml ORS/kg per 24 h for the 1^st ^10 kg of body weight^ ± ^	• 30-80 ml ORS/kg/h over 4 hours^ ± ^		
50 ml/kg/day for the next 10 kg^ ± ^	• 50-100 ml ORS/kg over 3-4 hours^#^*^$^		
20 ml/kg/day for remaining kg^ ± ^	• 50 ml ORS/kg over 4 hours^§^		
• 5 ml ORS/kg after each large watery stool^§^	• 10-25 ml ORS/kg/hour in 4 hours ^Φ^		
• 10 ml ORS/kg after each large watery stool ^Φ^			
	Maintenance^ ± §^		
Ongoing losses: ^$#^*	• 100 ml ORS/kg per 24 h for the 1^st ^10 kg of body weight		
• ORS^$^	50 ml ORS/kg/day for the next 10 kg	Refer to hospital	No evidence stated for ORS dosage ^$#^*^ ± § Φ^
• < 10 kg bodyweight: 60-120 ml ORS for each stool/vomit^#^*	20 ml ORS/kg/day for remaining kg^ ± ^		
> 10 kg bodyweight: 120-240 ml ORS for each stool/vomit^#^	• < 10 kg weight: 100 ml ORS/kg/day		
	10-20 kg weight: 1000 ml + 50 ml/kg for each kg > 10		
	20+kg weight: 1500 ml + 20 ml/kg for each kg > 20^§^		
			
	Ongoing losses: ^$#^*^§^		
	• Replace with ORS^$^		
	• 10 ml ORS/kg per stool/vomit ^ ± ^		
	• < 10 kg bodyweight: 60-120 ml ORS for each stool/vomit^#^*		
	> 10 kg bodyweight:120-240 ml ORS for each stool/vomit^#^		
	• 5 ml/kg ORS after each large watery stool^§^		
			

*WGO ^# ^CDC ^$^CPS ^ ± ^ARM

^∞^CCH ^Φ^NHG ^Ψ^ESP ^§^NICE

All guidelines state that breastfeeding should be continued throughout rehydration; an age-appropriate diet should be started during or after initial rehydration (4-6 hours) and dilution of the formula or the use of a milk-free formula is unnecessary (evidence based). These recommendations are based on a review of Brown et al. [53] and a large number of individual studies. Six guidelines state that the use of high-sugar beverages should be discouraged, six guidelines discourage the use of antiemetics and six state that antidiarrheals have no benefit or should not be used (all evidence based) (Table 3).

For the use of probiotics, the recommendations are inconsistent. The recommendations are based on 19 different studies and 5 of these studies, all meta-analyses [54-58], were used by more than one guideline. The systematic review by Allen et al. [54] was used by 4 guidelines (CCH, ESP, NHG and NICE). Guidelines in favor of probiotics (CCH, ESP and NHG) base their recommendations on studies with positive outcomes for different species of probiotics. The NICE guideline uses two of the previously mentioned studies [54,57], and adds studies without a positive result. They conclude that it is inappropriate to recommend probiotics despite some evidence for possible benefit. Because the conclusions in reported studies were not consistent and no separate analyses correcting for study setting (in- or outpatient) were performed in the meta-analyses used for the recommendations, the applicability to general practice remained inconclusive.

One recommendation was based on an outpatient population and is therefore generalizable to general practice. For all recommendations based on a mixed population, no separate analysis correcting for study setting was performed, therefore applicability remains inconclusive.

Table 5 presents additional recommendations based on consensus only, and provides information upon the number and the percentage of studies that corroborate for each consensus based recommendation. Four guidelines (CDC, ESP, NHG, NICE) recommend which patients should be seen by a physician. Consistent recommendations were made for the following risk factors: young age; high output; severe dehydration, persistent vomiting; and signs of severe cause for diarrhea or underlying disease. A consistent recommendation was made that blood tests and stool cultures should not be routinely performed. Six guidelines (Table 5) consistently recommended that a stool culture should be performed in sick patients with dysentery. Six guidelines consistently recommended that a child needed hospital reference or admittance, given social/logistic concerns; failure of initial rehydration; suspected serious alternative diagnosis and shock/severe dehydration. Three guidelines (CDC, CPS, NHG) consistently recommended that vomiting is not a contraindication for ORT. All other recommendations were not consistent.

**Table 5:** Recommendations on diagnosis and treatment based on consensus.

	Guidelines								
	**ARM**	**CDC**	**ESP**	**CPS**	**WGO**	**NHG**	**CCH**	**NICE**	
AGREE domain score 'rigour of development' (%)	88.1	35.7	90.5	38.1	28.6	66.7	85.7	88.1	
GP in guideline development team?	No	No	No	No	No	Yes	No	Yes	

**DIAGNOSIS**									**Consistent yes/no**

**Patients should be seen by physician if:**		$	$			$		$	**n = 4^#^**
They have risk factors for dehydration:									
Young age		+	+					+	Y^•^
Low birth weight/premature birth		+						+	N
Fever		+				+			N
Stopped breastfeeding								+	N
High output*		+	+			+		+	Y^•^
Persistent vomiting/>2 vomits per 24 h		+	+					+	Y^•^
Signs of malnutrition								+	N
Reported signs of severe dehydration		+				+		+	Y^•^
Fluid losses exceed intake						+			N
Not offered/able to tolerate supplemented fluids/suboptimal response		+						+	N
Signs of severe cause for diarrhea/underlying disease		+	+			+		+	Y^•^
Family circumstances		+						+	N
**Blood tests and stool cultures**	$	$	$		$	$	$	$	**n = 7^#^**
Should not be routinely performed	+	+	+		+	+	+	+	Y^•^
**Perform stool culture if:**	$	$	$		$	$		$	**n = 6^#^**
Dehydrated or febrile patients					+				N
Sick patients with dysentery ^¥^	+	+	+			+		+	Y^•^
Blood/pus in stool	+				+				N
Immuno-compromised patients			+			+		+	Y^•^
Patients with high infectivity for others						+			N
In case of outbreak			+						N
Recently traveled abroad	+					+		+	Y^•^
To verify another etiology/uncertain diagnosis/if no improvement after 7 days			+					+	N

**MANAGEMENT**									

**Refer/admit to hospital if**	$	$	$		$	$		$	**n = 6^#^**
Social/logistic concerns	+	+	+		+	+		+	Y^•^
Failure of initial rehydration	+	+	+		+	+		+	Y^•^
Suspected alternative serious diagnosis	+	+	+		+	+		+	Y^•^
Shock/severe dehydration		+	+		+	+			Y^•^
High risk of dehydration		+	+			+			N
Intractable/persistent vomiting		+							N
**Vomiting**		$		$		$			**n = 3^#^**
is not a contraindication for ORS		+		+		+			Y^•^
Do not give ORS if protracted vomiting despite small frequent feeding				+					N

§ : Guidelines making a recommendation on this subject

* = > 6 diarrheal stool in 24 h, or > 3 vomits in 24 h or watery diarrhea > 6 times a day > 3 days (<2 years: > 1 day)

^¥ ^Dysentery = diarrhea with mucus and/or blood in stool, with fever and abdominal pain

# = number of guidelines making a recommendation on this subject

+ = recommended

• = a general practitioner was present in at least one guideline production

For all consistent recommendations based on consensus, a GP was present in guideline development. They are therefore applicable to patients in general practice.

## Discussion

### Summary of the findings

Eight guidelines on the management of AGE in children were evaluated. Based on quality criteria as defined by the AGREE instrument, the reviewers recommended 4 guidelines for use in daily practice. There is a large variety in the classification of symptoms to different categories of dehydration. Of 14 signs and symptoms for assessing dehydration, 'decreased skin pinch' was the only sign mentioned in all guidelines. None of the signs were studied in general practice.

It is consistently recommended to use hypo-osmolar ORS to treat or prevent dehydration. This recommendation was based on evidence of good quality. The recommendations regarding ORS dosage, however, are inconsistent.

Except for probiotics, all recommendations on therapy are consistent.

### Quality of the guidelines

Recommendation for use in daily practice was mostly influenced by the items 'rigour of development', 'clarity and presentation', and 'editorial independence'. Guidelines receiving the highest scores on these items (ESP, NHG, CCH, NICE) received a 'recommend' or 'strongly recommend' rating.

Lowest scores were obtained for the items 'stakeholder involvement' and 'applicability'. This is in accordance with the findings of Alonso-Coello et al [59], who performed a systematic review of critical appraisal of guidelines in order to evaluate the quality of guidelines over time. They found that 'stakeholder involvement' and 'applicability' were two of the lowest scoring items of guidelines in general, and that little improvement was seen over two decades up to 2007. In contrast with their findings scores for 'rigour of development' were high for four of the guidelines we evaluated. Two of these were published after 2007 [33,37]. Alonso-Coello et al argue, that this domain may be the strongest indicator for quality, therefore our finding might indicate that some improvement on guideline development can be seen. Further improvement on stakeholder involvement and applicability as well as rigour of development might make guidelines for management of acute gastroenteritis in children more trustworthy to the user, and could improve guideline adherence.

### Quality of evidence

Three guidelines did not state the ranking system for the classification of the LofE of a recommendation. In addition, it was an intriguing finding that the evidence for a comparable recommendation could differ between guidelines, and vice versa that the recommendations could vary despite the same evidence. Quality of the evidence is an important indication for the strength of a recommendation, and should be consistently measured. We assume that non-adherence might be explained by the lack of consensus about quality of evidence, one of the most important indicators for the strength of a recommendation.

But obviously quality of evidence is not the only determinant of the strength of a recommendation. According to the GRADE Working Group[60,61], four key factors determine the strength of a recommendation: balance between desirable and undesirable effects; quality of evidence; values and preferences of the team, and costs. These additional arguments might have explained the heterogeneity between the recommendations. None of the guidelines, however, mentioned additional arguments that influenced their recommendations.

A uniform ranking system for the classification of the strength of recommendations is needed to make guideline-use more comprehensible and therewith increases adherence.

### Recommendations on diagnosis

The recommendations on signs related to dehydration are consistent, whereas the classification of signs in categories of dehydration is not. Even though hospital based evidence might, to some degree, be used for general practice, the generalizability of the recommendations for general practice is suboptimal. As a consequence, it remains unclear which signs are most important for classifying the extent of dehydration in a child with AGE, and to decide which child should be referred or admitted to a hospital.

### Recommendations on management

The recommended ORS regimen for each category of dehydration differed between guidelines. Possible reasons for this heterogeneity are the differences in cut-off points defining categories of dehydration, and the fact that expert opinions rather then evidence was used to formulate recommendations. As for now the optimal ORS regimen remains unclear.

The recommendations on prescribing probiotics are inconsistent and are evidently subject to interpretation by the guideline team. Only two guidelines (NHG, NICE) provide arguments for their interpretation of the available evidence, and conclude that there is insufficient evidence for recommendations on probiotics.

For none of the recommendations based on a mixed population it could be assessed if the study setting influenced the outcome, nor was this difference discussed in any guideline. For the guideline user, generalizability to general practice remains unknown.

### Limitations of the review

Appraisal of quality was performed by two reviewers, where preferably four appraisers are needed. Because we specifically wanted to review the guidelines from the perspective of the GP and both appraisers were GPs, ánd because the ranges of scores were small, we feel another two GPs would not give more precise or valid estimates of quality. Even though a thorough search of possible guidelines was performed, the possibility remains that we missed a guideline. We assumed that if a recommendation was based on consensus, because evidence was lacking, the presence of a GP gave the best available indication that applicability, and balance between benefit and harm had been evaluated from the perspective of the GP. We realise that this assumption is disputable.

## Conclusion

The present systematic review shows considerable variation in the quality of guidelines on AGE in children, as well as considerable inconsistencies between the recommendations. In 43% the quality of the evidence could not be interpreted because no level of evidence was stated and most recommendations are not generalizable to general practice. Although we know that ORT should be given, it remains unclear 'how' or to 'whom'; this may explain why adherence to treatment is currently suboptimal.

This study reveals implications for further research. Future studies, particularly in general practice, need to determine the value of clinical signs and symptoms in assessing dehydration. Second, the optimal ORS dosage for each grade of dehydration should be established, so that consistent recommendations can be made. Third, we need to investigate the considerations of clinicians to prescribe medication to children with AGE, and assess whether these considerations are valid. Finally, a uniform grading system, such as the GRADE system, should be used to grade the strength of guideline recommendations.

## List of abbreviations

AGE: Acute Gastroenteritis; AGREE: Appraisal of Guidelines for Research and Evaluation; ARM: Guideline by Armon et al; CCH: Guideline by Cincinnati Children's Hospital Medical Center; CDC: Centers for Disease Control and Prevention; CPS: Guideline by the Canadian Paediatric Society; ESP: Guideline by the European Society for Paediatric Gastroenterology, Hepatology and Nutrition and European Society for Paediatric Infectious Diseases; GP: General Practitioner; LofE- Level of Evidence; NHG: Guideline by the Nederlands Huisartsen Genootschap; NICE: Guideline by the National Institute for Health and Clinical Excellence; ORS: Oral Rehydration Solution; ORT: Oral Rehydration Therapy; WHO: World Health Organisation; WGO: Guideline by the World Gastroenterology Organisation.

## Competing interests

The authors declare that they have no competing interests.

## Authors' contributions

JB and MB contributed equally to this work. Both authors read and approved the final manuscript.

## Appendix 1

Search strings databases and researched websites

Pubmed

((child OR infant) OR ("Child"[Mesh]) OR ("Infant"[Mesh])) AND ((((guideline) OR (practice guideline) OR (consensus development) OR (recommendations)) OR ("Guideline "[Publication Type])) AND ((diarrhea OR diarr*) OR (("Diarrhea"[Mesh]) OR ("Gastroenteritis"[Mesh]))))

Embase

('diarrhea'/exp OR 'gastroenteritis'/exp OR gastroenteritis OR diarrhea) AND (guideline OR 'practice guideline'/exp OR consensus OR 'consensus'/exp OR recommendations) AND (child or 'child'/exp)

Cochrane

("Diarrhea"[Mesh]) OR ("Gastroenteritis"[Mesh]) AND guideline

Cinahl

((MH "Practice Guidelines") or guideline or practice guideline or recommendation or consensus) AND ((MH "Diarrhea") or (MH "Gastroenteritis") or diarrhea or gastroenteritis or diarr*) AND ((MH "Child") or child)

Websites: (diarrhea and/or gastroenteritis)

• http://www.guideline.gov (National Guideline Clearinghouse)

• http://www.tripdatabase.com (TRIP database)

• http://www.cma.ca (Canadian Medical Association)

• http://www.worldgastroenterology.org (World Gastroenterology Organisation)

• http://nhg.artsennet.nl/home.htm (Nederlands Huisartsen Genootschap)

• http://www.aap.org (American Academy of Pediatrics)

• http://www.who.int (World Health Organization)

• http://www.cdc.gov (Centers for Disease Control and Prevention)

• http://www.pediatriconcall.com

• http://www.library.nhs.uk (National Library for Health)

• http://www.emedicine.medscape.com

• http://www.childhealthfoundation.org (Child Health Foundation)

• http://www.racgp.org.au (The Royal Australian College of General Practitioners)

• http://www.rcgp.org.uk (Royal College of General Practitioners)

• http://www.nice.org.uk (National Institute for Health and Clinical Excellence)

• http://www.sign.ac.uk (Scottish Intercollegiate Guidelines Network (SIGN)

• http://clinicalevidence.bmj.com (BMJ Clinical Evidence)

• www.gfmer.ch (Geneva Foundation for Medical Education and Research)

• http://www.acponline.org (American College of Physicians)

• http://www.nzgg.org.nz/ (New Zealand Guidelines Group)

• www.cbo.nl (kwaliteitsinstituut voor de gezondheidszorg)

• www.nvk.pedianet.nl (Nederlandse Vereniging voor kindergeneeskunde)

• http://www.espghan.org (European Society for Peadiatric Gastroenterology, Hepatology and Nutrition

• http://www.g-i-n.net

## Appendix 2

The AGREE instrument is used to asses the quality of reporting and the quality of recommendations of a guideline. Quality is assessed on 23 items in six domains: scope and purpose; stakeholder involvement; rigour of development; clarity and presentation; applicability and editorial independence. A score for each item, ranging from 1 (strongly disagree) to 4 (strongly agree) is given by each appraiser after which a standardised domain score is calculated by summing all the scores of the individual items in a domain, and by standardising the total as a percentage of the maximum possible score for that domain. An overall assessment leads to recommendations for use of the appraised guideline.

For further information, see http://www.agreetrust.org

## Pre-publication history

The pre-publication history for this paper can be accessed here:

http://www.biomedcentral.com/1471-2296/12/134/prepub
